# True Precocious Puberty Following Treatment of a Leydig Cell Tumor: Two Case Reports and Literature Review

**DOI:** 10.3389/fped.2015.00093

**Published:** 2015-11-02

**Authors:** Alberto Verrotti, Laura Penta, Letizia Zenzeri, Laura Lucchetti, Paolo Giovenali, Pierpaolo De Feo

**Affiliations:** ^1^Department of Pediatrics, University of Perugia, Perugia, Italy; ^2^Department of Pathology, University of Perugia, Perugia, Italy; ^3^Department of Internal Medicine, University of Perugia, Perugia, Italy

**Keywords:** Leydig cell tumor, precocious pseudopuberty, true precocious puberty, GnRH stimulation test, GnRH analogs

## Abstract

Leydig cell testicular tumors are a rare cause of precocious pseudopuberty in boys. Surgery is the main therapy and shows good overall prognosis. The physical signs of precocious puberty are expected to disappear shortly after surgical removal of the mass. We report two children, 7.5 and 7.7 year-old boys, who underwent testis-sparing surgery for a Leydig cell testicular tumor causing precocious pseudopuberty. During follow-up, after an immediate clinical and laboratory regression, both boys presented signs of precocious puberty and ultimately developed central precocious puberty. They were successfully treated with gonadotropin-releasing hormone (GnRH) analogs. Only six other cases have been described regarding the development of central precocious puberty after successful treatment of a Leydig cell tumor causing precocious pseudopuberty. Gonadotropin-dependent precocious puberty should be considered in children treated for a Leydig cell tumor presenting persistent or recurrent physical signs of puberty activation. In such cases, therapy with GnRH analogs appears to be the most effective medical treatment.

## Introduction

Central precocious puberty is caused by premature activation of the hypothalamic–pituitary–gonadal axis, while precocious pseudopuberty results from peripheral over production of sex steroids (1–9). Leydig cell tumors are the most common hormone-secreting testicular tumors and are an unusual cause of precocious pseudopuberty in boys (5, 9). Surgical excision of the mass is usually curative with regression of signs, nevertheless the development of central precocious puberty after surgery can occasionally be observed (4–9). We report two boys who developed true precocious puberty after surgical treatment of a Leydig cell tumor of the testis that presented peripheral precocious puberty.

## Case Report

### Patient 1

**Figure 1:**
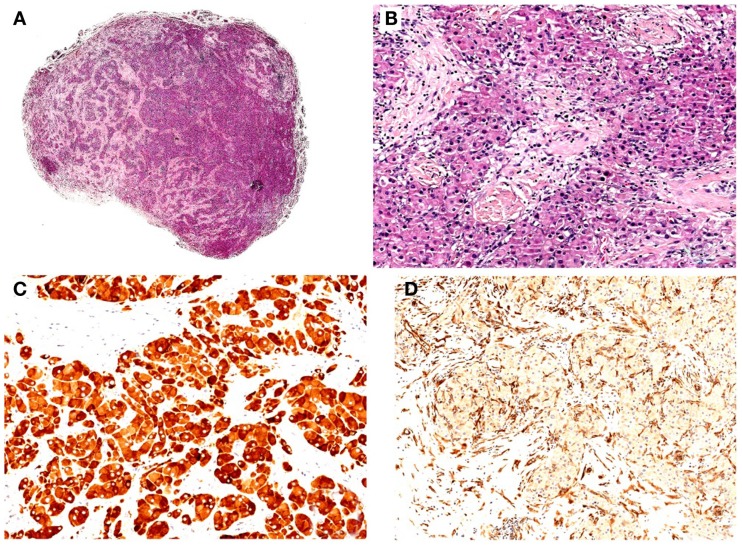
**(A)** Hematoxylin–eosin (H/E), original magnification 10x. Well-circumscribed nodule with solid/lobular architecture. **(B)** H/E, original magnification 200x. Neoplasm characterized by solid growth of polygonal cells with eosinophilic granular cytoplasm. No evidence of mitosis and/or necrosis. **(C)** IHC polymeric horseradish peroxidase (HRP)-linker antibody, original magnification 200x. Neoplastic cells are positive (brown) for gonadal hormone a-inhibin immunostain. **(D)** IHC polymeric horseradish peroxidase (HRP)-linker antibody, original magnification 200x. Neoplastic cells are negative for vimentin immunostain.

The patient was first evaluated at our Paediatric Endocrinology Department. Aged 7.5 years with a history of progressive appearance of secondary sexual characteristics for 4 months. He had pubic hair (Tanner stage PH2), enlarged testicular volume (Tanner stage G2: *left testis* 4 mL, *right testis* 6 mL) and an accelerated growth velocity. Pubertal stage has been performed according to Tanner-Marshall method on physical examination (1). Family history of precocious puberty was negative. His height was 134.6 cm (90th–97th centile, 1.63 SDS) and his body mass index (BMI) was 17.7 (1.17 SDS). Serum concentrations of follicle-stimulating hormone (FSH) and luteinizing hormone (LH) were suppressed, also after administration of gonadotropin-releasing hormone (GnRH) stimulation test. FSH and LH were measured by chemiluminescent immunoassay. His biological data at diagnosis are shown in Table 1. Tumor markers [alpha-fetoprotein (AFP), β-human chorionic gonadotropin (β-HCG)] were within the normal range. Adrenal function was normal. Ultrasound of the testis demonstrated an inhomogeneous hypoechogenic tumor located at the upper pole of the enlarged right testis (measuring 18.8 mm × 12 mm × 14 mm). Surgical enucleation of the testicular mass was performed. Histological examination revealed a Leydig cell tumor. Inhibin was expressed immunohistochemically and the index of proliferation (Ki67–MIB1) was <3% (Figure 1). Following surgery, levels of plasma sexual hormones rapidly returned to the normal prepuberal range and there was no sexual progression. Four months later, the patient presented with increased pubic hair (Tanner stage PH2) and increased bilateral testicular volume (Tanner stage G3: *left and right testis* 8 mL). Recurrent erections and ejaculations were observed. Bone age was accelerated to 12.5 years. Ultrasonographic examinations ruled out testicular tumor recurrence and brain magnetic resonance imaging (MRI) excluded a tumor of the hypothalamus or pituitary gland. The patient showed a pubertal response to GnRH stimulation test and the hormonal values are reported in Table 2. Therefore, non-organic central precocious puberty was diagnosed. Treatment with triptorelin (3.75 mg every 28 days) was started, resulting in clinical and laboratory regression; normal values of testosterone and normal, basal, and GnRH stimulated FSH and LH values were observed. At the last follow-up (2 years from the beginning of triptorelin therapy), the patient still continues treatment without adverse effects.

**Table 1:** **Hormonal assessment of boys with Leydig cell tumors at presentation and after surgery**.

Pt	Age (years)	Bone age G-P (years)	Testosterone (ng/dL)	Basal LH (mUI/mL)	Basal FSH (mUI/mL)	Peak LH^a^ (mUI/mL)	Peak FSH^a^ (mUI/mL)	Surgery	Testosterone values after surgery (ng/dL)	Reference
Pt 1	7.5	10.8	156	2	1.3	0.15	1.1	Testis-sparing	21	
Pt 2	7.7	10	183	1.5	0.8	1.2	0.9	Testis-sparing	19	
1	6.0	12	270	2.6	1.5	5.8	3.3	Orchidectomy	34	(4)
2	6.5	13	887	1.2	1	1.4	1.1	Orchidectomy	20.2	(5)
3	6.6	12	312	<0.07	<0.3	0.17	0.67	Orchidectomy	24	(6)
4	7.0	12.5	144	Low	Low	Low	Low	Orchidectomy	64	(7)
5	9.0	13.5	268	<0.1	<0.3	NR	NR	Orchidectomy	37	(8)
6	8.0	12	148	0.2	1.4	NR	NR	Testis-sparing	NR	(9)

*Pt, patient; G-P, Greulich and Pyle; LH, luteinizing hormone; FSH, follicle-stimulating hormone; NR, not reported*.

*^a^Maximum value after GnRH test (0.1 mg Relefact LHRH), testosterone normal value <20 ng/dL*.

**Table 2:** **Hormonal assessment of boys with secondary central precocious puberty after surgical treatment of Leydig cell tumors**.

Pt	Age (years)	Testosterone (ng/dL)	Basal LH (mUI/mL)	Basal FSH (mUI/mL)	Peak LH^a^ (mUI/mL)	Peak FSH^a^ (mUI/mL)	Therapy	Testosterone value after therapy (ng/dL)	Reference
Pt 1	7.8	178	3.2	4.5	24.1	10	Triptorelin	14	
Pt 2	8.0	210	3.8	4.8	19.4	10.7	Triptorelin	18	
1	6.5	280	12	11	57	19	Cyproterone acetate	34	(4)
2	6.7	141.3	11	5.5	22	13.5	Decapeptyl	20.2	(5)
3	7.7	47	0.67	4.53	20.48	12.73	leuprorelin acetate	16	(6)
4	7.1	201	NR	NR	Pubertal	Pubertal	leuprolide acetate	prepubertal	(7)
5	9.05	High	11.9	11.3	25.1	15.3	NR	NR	(8)
6	8.2	245	2.6	3.4	15.2	5.7	Triptorelin	<3	(9)

*Pt, patient; LH, luteinizing hormone; FSH, follicle-stimulating hormone; NR, not reported*.

*^a^Maximum value after GnRH test (0.1 mg Relefact LHRH), testosterone normal value <20 ng/dL*.

### Patient 2

The patient (aged 7.7 years) was evaluated because of progressive appearance of pubic hair and an accelerated growth velocity during the previous 6 months. He presented pubic hair (Tanner stage PH2) and an increased testicular volume (Tanner stage G2: *right testis* 4 mL, *left testis* 5 mL). Both testes were homogeneous and had no palpable masses. The penis was 12.5 cm long (10). Family history of precocious puberty was negative. His height was 140.2 cm (>97th centile, 2.31 SDS) and his BMI was 16.54 (0.62 SDS). His hormonal data indicated precocious pseudopuberty (see Table 1). Tumor markers (AFP, β-HCG) were within the normal range and adrenal function was normal. Ultrasound demonstrated an inhomogeneous hyperechogenic focal lesion located at the lower pole of the enlarged left testis (measuring 17 mm × 11.8 mm × 12 mm). Complete resection of the testicular mass was performed and the histological investigation revealed a Leydig cell tumor with immunohistochemical features of benignity. Testosterone serum levels declined to normal values (Table 1). Three months after surgery, the patient once again presented with increased pubic hair and increased bilateral testicular volume (Tanner stage G3: *left and right testis* 9 mL). The patient manifested increased erections and ejaculations. X-ray examination revealed a bone age of 12 years. Brain MRI ruled out a tumor of the hypothalamus or pituitary gland and ultrasound excluded a recurrence of the testicular mass. His hormonal values confirmed central precocious puberty (Table 2). Treatment with triptorelin (3.75 mg depot injections every 28 days) was started (Table 2). After 2 months, we observed clinical regression of physical signs. Currently, he continues therapy without adverse effects.

## Discussion

Leydig cell tumors are sex cord stromal tumors that arise from Leydig cells that produce testosterone (4, 9). Testicular tumors are very rare during childhood and represent only 1% of all pediatric solid tumors (11). Leydig cell tumors represent 3–6% of testicular masses in prepuberal males, even though they are the most common hormone-secreting testicular tumors (5, 7). Less than 25% have been described in boys aged between 5 and 10 years. These are mostly unilateral and benign although bilateral tumors have been described in 3–10% of cases and about 10% of the reported cases evolved into malignant neoplasms (5, 11). The clinical presentation of Leydig tumors is characterized by isosexual precocious pseudopuberty caused by increased production of androgens, mostly testosterone, and low gonadotropin levels. These patients develop secondary sexual characteristics in the absence of hypothalamic–pituitary activation (4–9). Previously, treatment for these lesions was a radical orchiectomy with lymphadenectomy when regional lymph nodes were involved (11). Early diagnosis of Leydig cell tumors allowed us to use conservative surgery as a first-line treatment (12). In the literature, several cases of males with Leydig cell tumors that developed isosexual precocious pseudopuberty are reported. In particular, Olivier et al. described a new case of a Leydig cell tumor in a boy and reported 23 other cases of boys with diagnosis of gonadotropin-independent precocious puberty secondary to a Leydig cell tumor from 1999 to 2012 (8). A similar prevalence of tumors between the right and left testes was observed and in all patients the histological analysis showed a Leydig cell tumor. (5, 6, 8).

Between 1998 and 2014, another 13 cases of boys with a Leydig cell tumor and precocious pseudopuberty were described (9, 13–22). In one case, the Leydig cell tumor had histological and immunohistochemical features of malignancy (16). The molecular analysis revealed a somatic activating mutation of the LH-receptor (replacement of aspartic acid with histidine at position 578: Asp578His) in only one patient (19). D’Alessio et al. reported a case of a metachronous contralateral Leydig cell tumor (22). Testis-sparing surgery was the first-line treatment (9, 13, 15, 17, 18, 20–22).

In our cases, the Leydig cell tumor had the typical clinical and hormonal characteristics of gonadotropin-independent precocious puberty (low LH and FSH with high testosterone). A testosterone-producing testicular tumor was identified as the cause of sexual precocity. It is interesting to underline that our patients developed gonadotropin-dependent precocious puberty, associated with accelerated growth and bone maturation, after surgical therapy. Only six other cases of central precocious puberty after initial treatment of a Leydig cell tumor, causing precocious pseudopuberty, have been described. This evolution may be more common than expected and should be considered in children with persistent or recurrent symptoms of precocious puberty after successful therapy. This phenomenon has also been observed in children with congenital adrenal hyperplasia, familial male precocious puberty, and McCune Albright syndrome (7). Although the pathogenesis of central precocious puberty after precocious pseudopuberty remains unknown, it is probable that early maturation of the hypothalamic GnRH pulse generator is caused by exposure to high concentrations of testosterone.

GnRH analog therapy is the most effective treatment for central precocious puberty. Progressive pubertal development should be documented over a 3- to 6-month period before GnRH analog treatment is initiated as this therapy is not always necessary. A surveillance period is useful to demonstrate the persistence of central stimulation and to detect patients that might benefit from treatment (3). However, in our cases, both patients presented with an accelerated skeletal maturation and rapidly progressive pubertal development (Tanner stage G3, erections and ejaculations) associated with a pubertal response to GnRH stimulation test. We, therefore, decided to eliminate the observational period and immediately start GnRH analog treatment.

In Tables 1 and 2, we summarized the clinical and hormonal features of our two cases and of the other six cases previously described.

In 1986, Criscuolo et al. presented a case of a 6-year-old boy with precocious pseudopuberty due to a Leydig cell tumor. After surgery, the hormonal profile progressively returned to the prepuberal range and sexual precocity disappeared. Six months later, the patient developed central precocious puberty, and was treated with cyproterone acetate (4). The second case was described in 2001: a 6.5-year-old boy with high levels of 17-hydroxyprogesterone that initially led to the erroneous diagnosis of congenital adrenal hyperplasia, and unilateral testicular enlargement due to a Leydig tumor. His physical findings and laboratory values did not improve with hydrocortisone but resolved after orchidectomy. Three months later, he presented with central precocious puberty and was initially treated with medroxyprogesterone and cyproterone acetate, and then with GnRH analogs (5). Kiepe et al. described a 6.6-year-old boy with sexual pseudo-precocity caused by a somatic activating mutation of the LH-receptor. One year after surgery, he developed a gonadotropin-dependent precocious puberty (6). Two other cases have been described in the literature, one in 2011 (7) and the other in 2012 (8).

The last case described (9) is an 8-year-old boy with isosexual precocity with gonadotropin hormone-independent testosterone hypersecretion. Ultrasound and histopathological analysis revealed a Leydig cell tumor. After surgery, testosterone levels remained high and hormonal examination showed gonadotropin-dependent precocious puberty. A long-acting GnRH analog was started and sexual precocity was suppressed (9).

## Conclusion

In conclusion, beside our two patients, only six other cases of central precocious puberty after successful treatment of a Leydig cell tumor, causing precocious pseudopuberty, have been described. We would like to underline that, even though it is a rare condition, gonadotropin-dependent precocious puberty should be considered in patients who have been successfully treated for a Leydig cell tumor and who present persistent or recurrent physical signs of sexual precocity. This can be caused by high levels of testosterone and other sex steroids that can trigger gonadotropin-dependent puberty.

GnRH analog treatment appears to be the most effective medical therapy available for progressive central precocious puberty.

## Conflict of Interest Statement

The authors declare that the research was conducted in the absence of any commercial or financial relationships that could be construed as a potential conflict of interest.
